# Li-Fraumeni syndrome presenting with de novo *TP53* mutation, severe phenotype and advanced paternal age: a case report

**DOI:** 10.1186/s13053-023-00272-2

**Published:** 2024-01-18

**Authors:** Juan Pablo Arango-Ibañez, Luis Gabriel Parra-Lara, Ángela R. Zambrano, Lisa Ximena Rodríguez-Rojas

**Affiliations:** 1 Facultad de Ciencias de la Salud, Universidad Icesi, Cali, Colombia; 2 Centro de Investigaciones Clínicas (CIC), Fundación Valle del Lili, Cali, Colombia; 3 Servicio de Hematología & Oncología Clínica, Departamento de Medicina Interna, Fundación Valle del Lili, Cali, Colombia; 4 Servicio de Genética, Fundación Valle del Lili, Cra. 98 #18-49, Cali, Valle del Cauca 760032 Colombia

**Keywords:** Li Fraumeni syndrome, *TP53*, P53, De novo mutation, Advanced paternal age, Severe phenotype

## Abstract

**Background:**

Li-Fraumeni syndrome (LFS) is an autosomal dominant hereditary cancer syndrome caused by pathogenic variants in the gene *TP53*. This gene codes for the P53 protein, a crucial player in genomic stability, which functions as a tumor suppressor gene. Individuals with LFS frequently develop multiple primary tumors at a young age, such as soft tissue sarcomas, breast cancer, and brain tumors.

**Case presentation:**

A 38 years-old female with a history of femur osteosarcoma, ductal carcinoma of the breast, high-grade breast sarcoma, pleomorphic sarcoma of the left upper limb, infiltrating lobular carcinoma of the breast, gastric adenocarcinoma, leiomyosarcoma of the right upper limb, and high-grade pleomorphic renal sarcoma. Complete molecular sequencing of the *TP53* gene showed c.586 C > T (p.R196X) in exon 6, which is a nonsense mutation that produces a shorter and malfunctioning P53. Family history includes advanced father’s age at the time of conception (75 years), which has been associated with an increased risk of de novo germline mutations. The patient had seven paternal half-siblings with no cancer history. The patient received multiple treatments including surgery, systemic therapy, and radiotherapy, but died at the age of 38.

**Conclusions:**

Advanced paternal age is a risk factor to consider when hereditary cancer syndrome is suspected. Early detection of hereditary cancer syndromes and their multi-disciplinary surveillance and treatment is important to improve clinical outcomes for these patients. Further investigation of the relationship between the pathogenic variant of *TP53* and its phenotype may guide the stratification of surveillance and treatment.

## Background

Li-Fraumeni syndrome (LFS) is a hereditary cancer syndrome with an autosomal dominant pattern of inheritance and causes multiple types of tumors, including a wide range of women’s cancers [1]. This disease is caused by pathogenic variants of the *TP53* gene (MIM*191,170) in chromosome 17p13, which codes for the protein P53, one of the most important proteins involved in carcinogenesis regulation. Up to 50% of all cancer types are found to have a pathogenic variant of P53 [2]. *TP53* is implicated in genetic stability, apoptosis, and inhibition of angiogenesis. Variants causing an abnormal activity of the protein may lead to cancer, which exhibits its role as a tumor suppressor gene. Common cancers found in patients with LFS include soft tissue sarcomas, breast tumors, leukemias, and adrenal cancer [3].

De novo mutations are responsible for a considerable number of cases of LFS. They result from mutations in any stage of gametogenesis and are associated with severe phenotypes of disease because of lesser exposure to natural selection. These mutations are also associated with advanced paternal age, which is explained by accumulated mutations in older germline stem cells, especially in males due to a higher cell division compared to female germline stem cells. This is reflected in single nucleotide mutations of a gene, which produce a pathogenic protein [4, 5].

The case of a patient diagnosed with LFS with a suspected de novo mutation and associated with advanced paternal age is presented below.

## Case presentation

A 38-year-old woman with a history of eight neoplasms. Her family history included advanced paternal age (75 years old) at conception and prostate cancer in the father’s eighties. At the time of the patient’s birth, the mother was 23 years old, and she had an unremarkable medical history. There was no history of early-onset cancer in other family members.

The patient’s initial tumor was an osteosarcoma of the right femur, diagnosed at the age of 12. The tumor was treated with neoadjuvant chemotherapy comprising cisplatin-doxorubicin and subsequent surgical resection. Chemotherapy was continued for six months after resection.

At the age of 26, the patient presented with a rapidly growing mass in the right breast, which was diagnosed as synchronous ductal carcinoma (pT2 pN0 MX) with positive estrogen and progesterone receptor expression, negative HER2 status, and high-grade sarcoma of the breast (pT2 pN0 MX). A radical mastectomy was performed, followed by chemotherapy (tamoxifen, goserelin, and ibandronic acid) for five years.

One year later, doxorubicin-induced cardiomyopathy was diagnosed and the patient was admitted. During this hospitalization, a left epitrochlear mass was observed and removed. Pathology reported a high-grade pleomorphic sarcoma (pT1 pN0 M0) with free margins of resection. At this point, LFS was clinically diagnosed with the Chompret Criteria. A comprehensive examination of the patient’s family cancer history revealed that none of the seven paternal half-siblings had a history of cancer (the oldest was 68 years old at this point). The patient’s father had been diagnosed with prostate cancer and died at the age of 82. Neither the patient’s mother nor her only maternal uncle had a history of cancer.

At the age of 28, the patient was found to have another mass in her left breast. The pathology report showed invasive lobular carcinoma (pT2 pN0 MX). Consequently, she underwent a radical left mastectomy. At the age of 30, the patient underwent sequencing for *TP53* from a peripheral blood sample which revealed the pathogenic variant c.586 C > T (p.R196X) in exon 6; thus, LFS was confirmed. The patient refused routine surveillance after diagnosis due to personal reasons.

At 35 years of age, an ovarian mass was incidentally detected during routine transvaginal echography. Subsequent evaluation with abdominopelvic contrasted computed tomography revealed a right adnexal mass, peritoneal carcinomatosis, and abdominal adenomegalies. Further examination via upper endoscopy detected an ulcerated mass in the lesser curvature of the stomach, which biopsies confirmed as infiltrating gastric adenocarcinoma (pT2 N0 M1) positive for *HER2* and *Helicobacter pylori*. Colonoscopy showed no abnormal findings. The ovarian tumor was resected, along with a hysterectomy, bilateral salpingo-oophorectomy, appendicectomy, and omentectomy performed laparoscopically. Pathological examination confirmed metastatic signet ring cell adenocarcinoma, likely from a gastrointestinal source. A diagnosis of stage IV diffuse gastric adenocarcinoma with bilateral Krukenberg tumors and lymphatic and peritoneal metastasis was made. One month after surgery, the patient was started on docetaxel-carboplatin-fluorouracil (TPF) and trastuzumab.

During hospitalization for chemotherapy, the patient presented with a right epitrochlear mass that rapidly increased in size. Surgical excision was performed, and the pathology exam revealed a stage II leiomyosarcoma (pT2, pN0, M0) with compromised margins. A positron emission tomography (PET) scan showed a hypermetabolic lesion in the right upper limb region where tumor resection was performed and a nodular hypermetabolic lesion anterior to the left kidney, which was thought to be a peritoneal implant of gastric adenocarcinoma. The medical board opted to continue monitoring these lesions, given the patient’s ongoing chemotherapy treatment.

Five months after the diagnosis of gastric adenocarcinoma the patient completed six cycles of docetaxel + cisplatin + 5-fluorouracil (DCF)-trastuzumab therapy and underwent a complete cytoreduction surgery with Hyperthermic Intraperitoneal Chemotherapy (HIPEC). A follow-up complete body PET scan showed an increased size of the mass previously seen in the left kidney, now compromising the renal vein; other findings included a solitary pulmonary nodule in the right lung and hypermetabolic internal mammary nodules suspicious of metastasis of gastric adenocarcinoma; no hypermetabolic lesions were seen in the gastrointestinal apparatus or limbs. A radical nephrectomy was performed with a pathology reporting a high-grade pleomorphic sarcoma with an invasion of renal sinus, perihilar region, and adjacent fat tissue with compromised margins (pT2b pN0 M0).

Somatic genomic profiling (Foundation Medicine Inc., Cambridge, MA, USA) was performed using a biopsy of high-grade pleomorphic sarcoma of the kidney to assess possible therapeutic targets. The results showed *EED* rearrangement in exon 6, *RB1* rearrangement in intron 17, and TP53 R196*. Biomarker findings exhibited stable microsatellite status and tumor mutational burden 5 Muts/Mb. Other variants with unknown significance found in the patient’s tumor included *APH1A* amplification, *BCOR E1708D*, *CDKN2C* amplification, *FGF10* amplification, *FGF19 S78C*, *IL7R* amplification, *LRP1B V4109I*, *LRRK2 M379I*, and *Q1657L*, *MLL2 L2161H*, *RICTOR* amplification, *SETD2 H1284L*, *SPEN K2065E*, *TNFRSF11A* splice site *730 + 1G > A*, and *ZNF217 M410V*.

Following the patient’s nephrectomy, a medical board convened to discuss treatment options. The patient declined targeted cancer therapy and opted to continue chemotherapy instead. Three months later, the patient developed thrombosis in multiple veins, including the right renal vein, both common iliac veins, and the inferior vena cava, as well as a pulmonary embolism. Despite medical intervention, the patient died at the age of 38.

The summary of each neoplasm is shown in Table 1. All tumors were diagnosed histologically.

**Table 1:** Description of patient’s tumors

Morphology	Topography	Age at diagnosis	Clinical staging	Treatment
Osteosarcoma	Right femur	12	*	Surgery (distal femur resection) and chemotherapy
Ductal carcinoma in situ	Right breast	26	0 (pT2 pN0 MX)	Surgery (radical mastectomy)
High-grade stromal sarcoma	Right breast	26	0 (pT2 pN0 MX)	Surgery (radical mastectomy) and chemotherapy
High-grade pleomorphic sarcoma	Left upper limb (epitrochlear)	27	0 (pT1 pN0 M0)	Surgery (excision)
Invasive lobular carcinoma	Left breast	28	0 (pT2 pN0 MX)	Surgery (radical mastectomy)
Diffuse gastric adenocarcinoma with bilateral Krukenberg tumors	Stomach (lesser curvature)	35	IV (pT2 pN0 M1)	Surgery (cytoreduction, radical gastrectomy, Sugarbaker procedure)
Leiomyosarcoma	Right upper limb (epitrochlear)	35	II (pT2, pN0, M0)	Surgery (excision) and radiotherapy
High-grade pleomorphic sarcoma	Left kidney	37	II (pT2b pN0 M0)	Surgery (radical nephrectomy)

*No data

## Discussion

Over 500 families with LFS have been reported in the literature [6]. Around 10-20% of cases of LFS have been linked to de novo mutations and 80% are traced to the paternal allele [7, 8]. The main risk factor for de novo mutations is advanced paternal age, which is attributed to higher rates of cell division in male germline stem cells and a longer period of reproductive capability compared to females. In addition, pathogenic de novo mutations in male germline stem cells can provide selective advantages such as clonal expansion. These pathogenic variants are called “selfish mutations” because they confer a selective advantage but are harmful to the offspring, a phenomenon also observed in cancer [8].

Consequently, a pathogenic variant of the *TP53* gene in paternal germline stem cells may confer a positive selective pressure, increasing the likelihood of the variant being passed down to offspring. Considering the lack of early-onset cancer in the patient’s father, mother, and half-siblings, along with the advanced paternal age at conception (75 years), we strongly suspect that a de novo pathogenic variant is highly likely to be the cause of the patient’s severe LFS phenotype. Thus, when evaluating patients with suspected hereditary cancer syndromes, it is important to consider advanced paternal age as a significant risk factor and should be identified during genetic counseling. Another potential explanation could be incomplete penetrance; LFS is associated with a lifetime cancer risk of 70% or higher in men and 90% or higher in women [3, 9]. This might offer insights into the patient’s father’s clinical history of absent features of LFS. However, this explanation appears less probable, as there is a significant number of paternal half-siblings with no documented history of cancer.

The morphology and location of this patient’s cancers were consistent with a classic presentation of LFS: breast cancer and soft tissue sarcomas [5]. Although LFS usually presents with *HER2*-positive breast cancer, as seen in this patient, up to 40% of patients present with *HER2*-negative breast cancer [10]. The severe phenotype in this patient is suggested by the high number of primary malignancies (eight) and the age of onset. One study of 322 patients with pathogenic variants of *TP53* found that 43% developed multiple malignancies and less than 1% more than six primary tumors. Moreover, the mean onset of cancer reported was 24.9 years, which is 12 years older than the onset in this patient. Contrary to previous findings, the presence of a severe phenotype in this case caused by a nonsense pathogenic variant contradicts the commonly observed milder phenotypes associated with loss-of-function mutations such as nonsense mutations, frameshift mutations, or genomic rearrangements, in comparison to dominant negative missense mutations in terms of disease onset [5, 10]. The observed phenomenon may be attributed to the complex interactions between P53 and other molecules, as well as exposure to environmental factors that can cause genotoxic effects.

The c.586C > T (p.R196X) nonsense mutation has been previously reported and is categorized in ClinVar as a pathogenic variant. One study found it as a de novo mutation in a male patient who developed rhabdomyosarcoma by 12 months [11]. Another study reported this variant in a female patient who developed liposarcoma and two breast cancers at 32, 35, and 37 years of age respectively [12]. This pathogenic variant was also reported in a female patient who had colonic adenoma and intraductal carcinoma of the breast at 35 and 37 years respectively [13]. A comparative table of the mentioned cases, including the patient in our case, is provided in Table 2. The first two cases described previously, along with our case, suggest that the nonsense mutation c.586C > T (p.R196X) has a high pathogenicity regarding disease onset or the number of primary tumors. However, the last case presented did not exhibit such high pathogenicity. All four cases exhibit tumor morphologies typically seen in LFS patients. Our case is consistent with a recent study that found individuals with germline *TP53* pathogenic variants leading to a loss of p53 function exhibited a more severe clinical phenotype compared to those with variants causing partial P53 deficiency [14]. Reporting more cases on the genotype-phenotype relationship, and the phenotype of germline pathogenic variants can significantly enhance our understanding of LFS. As current guidelines suggest, this knowledge can help classify ‘high-cancer-risk’ and ‘low-cancer-risk’ genotypes, which could be used in the future to stratify patient surveillance and treatment. This interpretation should be performed carefully by experts, and considering consultant agencies, such as American College of Medical Genetics (ACMG), could be adequate [15, 16].

The patient, in this case, declined surveillance for personal reasons, which may account for the advanced stage of the diagnosis of some tumors. Current clinical guidelines for LFS recommend the surveillance using the Toronto Protocol, which includes for adults a full physical check-up every 6 months, breast magnetic resonance imaging (MRI) every year from 20 to 75 years, considering risk-reducing mastectomy, brain MRI every year, whole-body MRI every year, abdominal and pelvic ultrasound every year, and upper and lower gastrointestinal endoscopy every two to five years [15, 16]. The use of chemotherapy in this patient at a young age could have been implicated in the development of subsequent primary tumors. Treatment with radiotherapy or chemotherapy in patients with LFS must be carefully evaluated by specialists due to the increased risk of developing other primary tumors because of genotoxic effects, especially in patients with germline mutations. Hence, surgical treatments or non-genotoxic chemotherapies should be prioritized [5, 15]. Early detection of patients with LFS and their multidisciplinary surveillance and management is crucial for improving clinical outcomes.

### Limitations

Confirmation of paternity or the investigation of the pathogenic variant in the parents was not feasible, as they had deceased prior to the patient’s medical management.

## Conclusion

Advanced paternal age is an important risk factor to consider when hereditary cancer syndromes are suspected due to its association with de novo mutations. These mutations may manifest as a severe phenotype, as evidenced in this case, where a pathogenic nonsense mutation resulted in the loss of P53 function. More studies are needed to assess the genotype-phenotype relationship in patients with LFS, which could aid in the stratification of their surveillance and treatment. Treatment radiotherapy or chemotherapy in patients with LFS must be carefully evaluated and surgical treatment and non-genotoxic chemotherapy should be prioritized. Early detection of hereditary cancer syndromes and their multidisciplinary management are crucial for improving clinical outcomes.

## Appendix

**Table 2:** Description of patients with c.586 C > T (p.R196X) *TP53* pathogenic variant

Case	Age at diagnosis of first tumor (years)	Age at death or age at end of follow-up (years)	Gender	Tumor description
Present case (2023) Colombia	12	38 (death)	Female	Femur osteosarcoma, ductal carcinoma of the breast, high-grade breast sarcoma, pleomorphic sarcoma of the left upper limb, infiltrating lobular carcinoma of the breast, gastric adenocarcinoma, leiomyosarcoma of the right upper limb, high-grade pleomorphic renal sarcoma
Bendig, *et al.* [11] Germany	1	1 (follow-up)	Male	Rhabdomyosarcoma
Vahteristo, *et al.* [12] Finland	32	37 (follow-up)	Female	Liposarcoma, breast cancer
Villani, *et al. *[13] Canada	35	39 (follow-up)	Female	Colonic adenoma, intraductal carcinoma of the breast

## Data Availability

Not applicable.
